# Additive interactions of nanoparticulate ZnO with copper, manganese and iron in *Pisum sativum* L., a hydroponic study

**DOI:** 10.1038/s41598-020-70303-8

**Published:** 2020-08-11

**Authors:** Elżbieta Skiba, Sylwia Michlewska, Monika Pietrzak, Wojciech M. Wolf

**Affiliations:** 1 Institute of General and Ecological Chemistry, Lodz University of Technology, Zeromskiego 116, 90-924 Lodz, Poland; 2 Laboratory of Microscopic Imaging and Specialized Biological Techniques, Faculty of Biology and Environmental Protection, University of Lodz, Banacha 12/16, 90-237 Lodz, Poland

**Keywords:** Chemical biology, Plant sciences, Environmental sciences, Chemistry, Nanoscience and technology

## Abstract

Widespread occurrence of ZnO nanoparticles in environment follows the growing number of applications either in technology or agriculture. The impact of five forms of nanoparticulate ZnO on copper, manganese and iron uptake by *Pisum sativum* L. cultivated in Hoagland solutions was investigated. Plants were collected after twelve days of zinc administration. Effect of bulk ZnO has also been studied. Initial zinc concentration was 100 mg L^−1^. Nanoparticles were characterized by the Transmission Electron Microscopy, Dynamic Light Scattering and Zeta potential measurements. Metal contents were analyzed using the Atomic Absorption Spectrometry with flame atomization for samples digested in a microwave closed system. Analysis of variance indicated that zinc species at either molecular or nanoscale levels altered Cu, Mn and Fe uptake and their further transport in pea plants. In particular, significant reduction of Mn and Fe combined with the Cu increase was observed. Additive interactions originated by nanoparticles affect the heavy metals uptake and indicate pollutants migration pathways in plants. Unfortunately, regulations for the plant cultivation were formulated when anthropogenic nanoparticles were not in common use. They underestimate complexity of metals interactions in either plant or habitat. Our results indicate that these additive interactions cannot be neglected and deserve further investigations.

## Introduction

Green pea (*Pisum sativum* L.) is one of the most extensively cultivated grain legumes worldwide. Plants are well adapted to diverse soil zones in either cool or mild climatic regions^1^. Their seeds are rich in proteins, carbohydrates, dietary fibers, vitamins as well as minerals and are commonly used as vegetable or important protein source. The latter is of particular relevance when animal feed is concerned^2^. The global production of pea is steadily growing as indicated by the projected Compound Annual Growth Rate (CAGR) of 5.9% and has approached 20 million tonnes in 2018^3^.

Pea genetics was thoroughly studied and in combination with better plant breeding methods has led to variety of improved plant species^4^. Nowadays, pea is an important non model plant widely used in applied system biology studies^5^. The pea genome has not been completely determined as yet. Nevertheless, it is being frequently applied as a model plant with the almost complete transcript coverage^6^.

The impact of nanoparticles (NPs) on plants physiology and their nutritional quality is usually assessed using two leading methodologies as presented by Jośko and Oleszczuk^7^. Initially, the long-term growth in soils supplemented with representative concentrations of investigated nanoparticles was applied^8,9^. Nowadays, the soilless plant cultivation is gaining increasing popularity^10^. Hydroponic techniques promote plant growth in nutrient solutions. Their usage by far exceeds the laboratory scale and they have found numerous applications in commercial crop production^11^. Several advantages of hydroponic cultures are highly appreciated, i.e. the soil sterilization can be skipped, plant diseases are better controlled, nutrient administration is easier and more accurate while separation of root material is possible without damaging the root hairs. Plant samples harvested from liquid solutions are more uniform leading to the statistically sound results^12^. Notable, physiological processes can be observed in a more comprehensive way^13,14^. However, as pointed out by Rastogi et al.^15^ and Zhao et al.^16^, responses of plants grown in hydroponic media may not be the same as observed in soil conditions.

The influence of ZnO nanomaterials on plants is commonly referred to the direct contact of NPs with plant tissues with special emphasis on possible interactions of solvated zinc ions and the reactive oxygen species mediated processes^17^. The latter mechanisms are complex and far from being thoroughly understood. As pointed out by Abbas et al.^18^, Dwivedi et al.^19^ as well as by Judy and Berstch^20^, the phytotoxicity of particular nanomaterial depends on its physical and chemical properties. The nanoparticles size, their surface topology and dynamics of aggregation are among the most important^21^. The influence of bare and hybrid ZnO NPs on green pea plants as cultivated in soil environment were studied by García-Gómez et al.^22^ and Mukherjee et al.^9,23,24^. However, to the best of knowledge none investigations on combined, additive interactions of essential heavy metals in *Pisum sativum* plant grown in hydroponic media have been reported so far.

Nowadays, the growth of nanomaterials production and usage is widespread indeed. The generally acknowledged forecast published by the Allied Market Research predicts that the value of the nanomaterials global market will approach 55 billion USD in 2022^25^. Special attention is paid to the metal and metal oxide nanoparticles^26^. In particular, zinc oxide based nanoparticles exhibit very unique chemical and physical properties and found diverse applications as multifunctional nanomaterials^27^. Especially, the antibacterial activity of ZnO NPs induced numerous applications in pharmaceutical industry^28^. Substantial UV radiation absorption ability prompts their usage as essential component of various cosmetic products^29^. They are also applied in rubber industry as an important crosslinking agent mainly for advanced tires production^30^. According to the well documented review of Piccinno et al.^31^ nanoparticulate ZnO occupies third position on the market of manufactured metal nanoparticles with production approaching 550 tonnes annually. On the other hand, Keller et al.^32^ concluded that ZnO NPs synthesis is blooming and in 2010 yielded over 30,000 tonnes of ZnO nanomaterials per year. The ZnO nanomaterials are mostly produced by large renown manufacturers. Unfortunately, they tend to treat exact market data as a classified material. However, the current level of production and usage raises the question of possible environmental impact of ZnO nanoparticles at either local or global level. The thorough reviews of the subject were recently published by Gupta and Shwarma^33^ and Baddar et al.^34^ who simultaneously pointed out the urgent need of extensive studies in the subject.

Zinc deficiency is recognized as a global nutritional and health problem^35^. In more than 30% of the world’s agricultural land zinc concentration is insufficient^36^ with the 17% of the overall human population being affected^37^. Contemporary breeding and agronomic approaches tend to alleviate this issue. Biofortification of zinc requires the thoroughly selected fertilizers^38^ with carefully controlled solubility and bioavailability. Nanotechnology offers remarkable opportunities in this area as was recently pointed out by Bala et al.^39^ in their work on zinc fortification in rice cultivated under low Zn concentration. In particular, the ZnO NPs influence on the Cd uptake by plants was recognized by Ali et al.^40^. Strong impact of ZnO NPs on the wheat yield and the Cd concentration in grain was identified by Khan et al.^41^. They discovered that concentration of 100 mg kg^−1^ ZnO NPs was the most effective in curbing the Cd transfer from roots to grain. Substantial additive Zn–Cd effect was also recognized for *Melissa officinalis.* The Zn uptake and accumulation in either roots or above-ground parts in this plant was inversely proportional to the cadmium concentration in soil^42^.

In this work we investigate several types of nanoparticulate and bulk zinc oxides. Its goal is aimed at foundations of copper, manganese and iron uptake by *Pisum sativum* L. cultivated in hydroponic solution. Special emphasis is put on plant growth parameters and additive interactions originated by above micronutrients. The latter effects are rarely acknowledged in agriculture and obviously deserved further studies.

## Methods

### Zinc compounds

**Figure 1:**
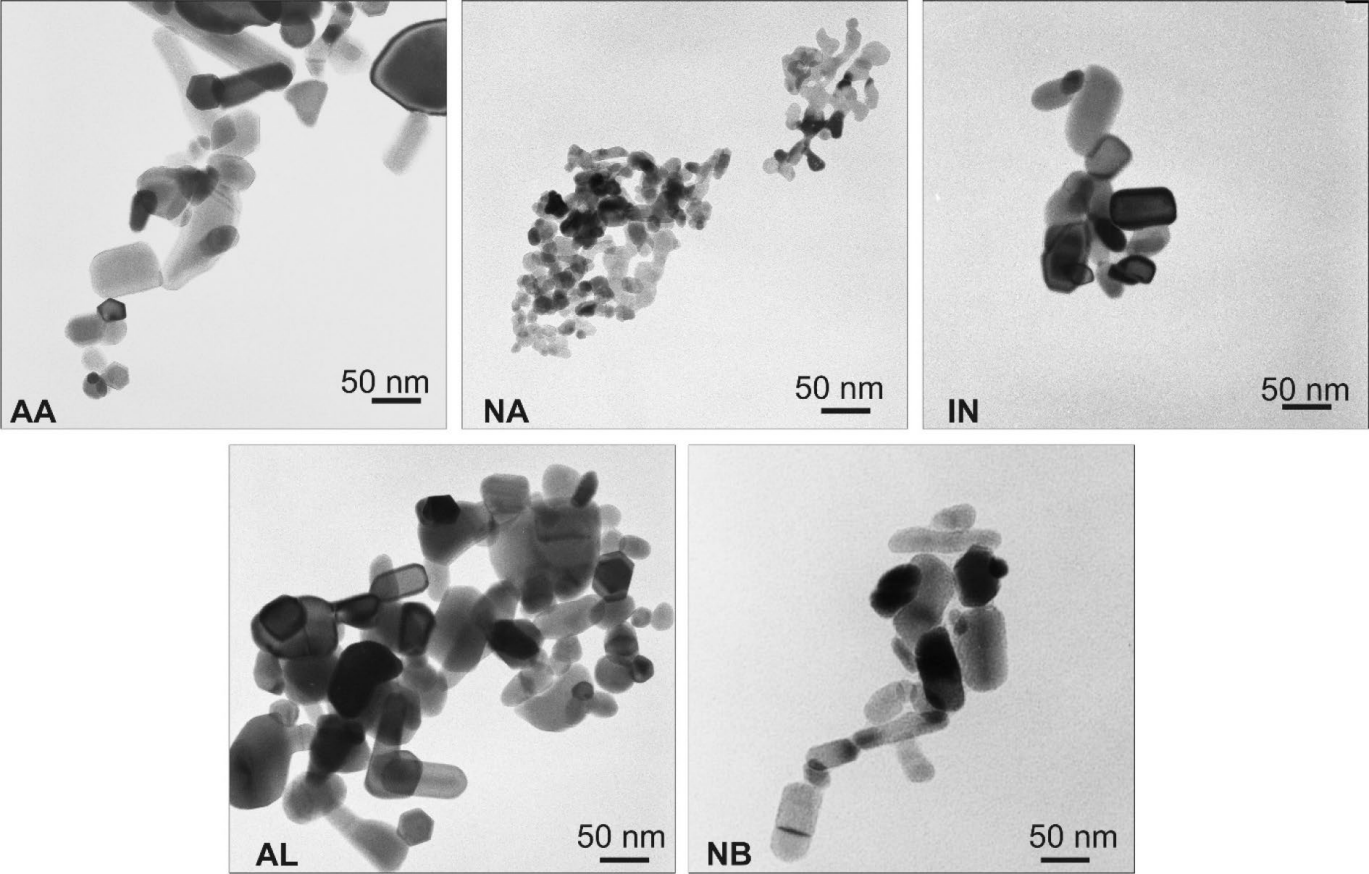
Transmission electron microscopy (TEM) images of zinc oxide nanoparticles.

ZnO (99.995%) in a bulk (BU), wurtzite hexagonal structure (as confirmed by the powder X-ray diffraction technique) and five types of commercially available zinc oxide nanoparticles were used in the experiment. They were obtained from Alfa Aesar, Nanostructured & Amorphous Materials Inc., Intrinsiq Materials, Sigma Aldrich and Byk, Additives & Instruments and are further abbreviated as AA, NA, IN, AL and NB, respectively. The first four formulations were powders while the last one was obtained from the manufacturer as a liquid dispersion. All substances were used without further purification. The shape, size and structure of zinc oxide nanoparticles were investigated by transmission electron microscopy^43,44^. Ten microliters of particular sample solution was placed on the 200-mesh copper grid coated with the carbon surface, washed in demineralized water and dried at room temperature. Images were collected with the JEOL-1010 instrument (Fig. 1). The ZnO nanoparticles dissolution in growing media was determined by two steps procedure as described by Landa et al.^45^ and Mukherjee et al.^24^. The appropriate amounts of the zinc oxide (AA, NA, IN, AL and NB) were added to 100 mL volumetric flasks in order to obtain 100 mg L^−1^ zinc concentration. Suspensions were centrifugated at 14,000 rpm for 2 h. Supernatants were collected, filtrated through the 0.2 µm filter and zinc concentrations were determined using the ICP-OES instrument (Analytik Jena, Jena, Germany). Zeta Potentials and hydrodynamic sizes of NPs were collected with Zetasizer Nano (Malvern Instruments Ltd., UK) in raw solutions with zinc concentration 100 mg L^−1^. All measurements were replicated three times. Average TEM and hydrodynamic particle sizes augmented with zeta potentials are summarized in Supplementary Information Table S1.

### Experimental setup

Iłówiecki sugar pea (*Pisum sativum* L.) quality class A seeds from „PNOS” Co. Ltd., Ożarów Mazowiecki were used in the study. Seven series, each constituted of six pots with 26 plants were used. Seeds were surface sterilized with 70% ethanol for 10 min, and washed carefully with demineralized water. They were placed on a moderately wet filter paper in Petri dishes to germinate in a dark for 3 days at 22 °C. At that point, their mean stage of growth was 09 according to the BBCH scale^46^. Next, seedlings were grown for 4 days at 21 °C in aerated Hoagland solution: KNO_3_ (0.51 g L^−1^), Ca(NO_3_)_2_·4H_2_O (1.18 g L^−1^), MgSO_4_·7H_2_O (0.49 g L^−1^), KH_2_PO_4_ (0.14 g L^−1^), H_3_BO_3_ (0.6 mg L^−1^), MnCl_2_·4H_2_O (0.4 mg L^−1^), ZnSO_4_·7H_2_O (0.05 mg L^−1^), CuSO_4_·5H_2_O (0.05 mg L^−1^), FeEDTA (10.28 mg L^−1^) and Na_2_MoO_4_·2H_2_O (0.02 mg L^−1^) at pH 5.9. The intensity of radiation was 170 μE m^−2^ s^−1^, 16/8 h day/night photoperiod was used and the growth medium replacement period was 48 h.

Later, six series were administered with 750 mL of aerated Hoagland solution per pot and augmented with 100 mg L^−1^ of Zn in the form BU, AA, NA, IN, AL and NB, respectively. The metal dose was adjusted to affect plants physiology but not to be lethal for the pea plant. ZnO NPs stock solutions were sonicated for 30 min in ultrasonic bath Sono Swiss SW 6H.

**Figure 2:**
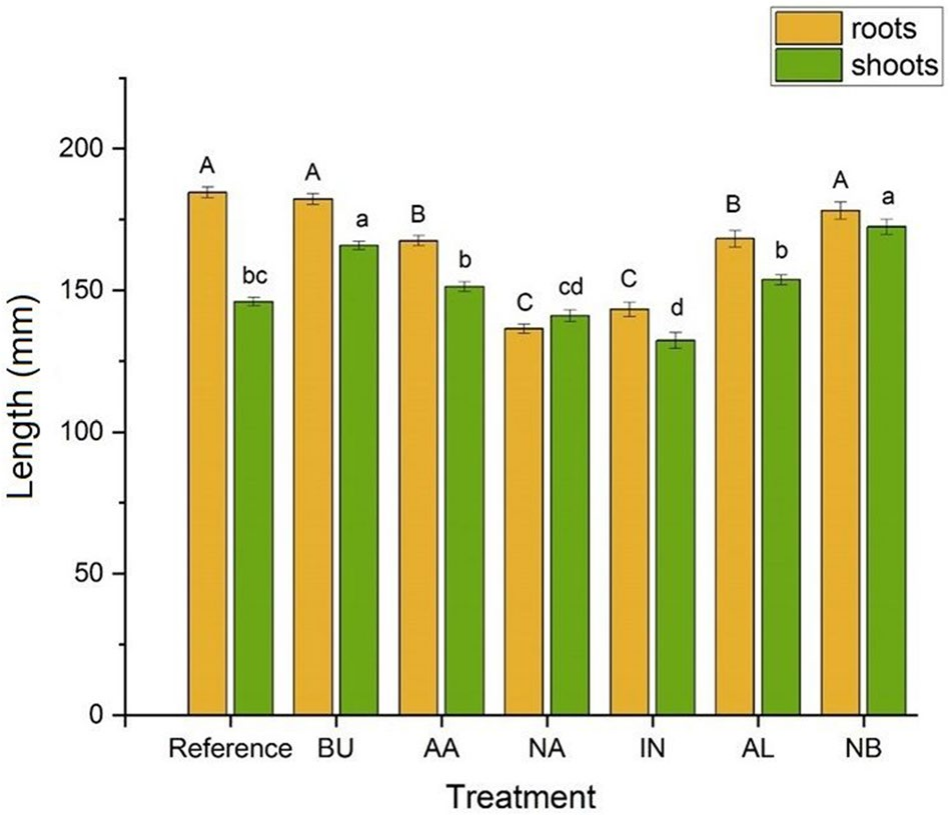
Root and stem lengths (mm) of sugar pea plants after 12 days of contact with ZnO species. Values are means over all plants in particular treatment. Roots are indicated in yellow while shoots are in green. Vertical bars represent standard deviations. Distinct letters show the statistically significant difference as calculated with the Tukey’s HSD test, roots and stems are treated separately. The significance level α = 0.05 was applied.

**Figure 3:**
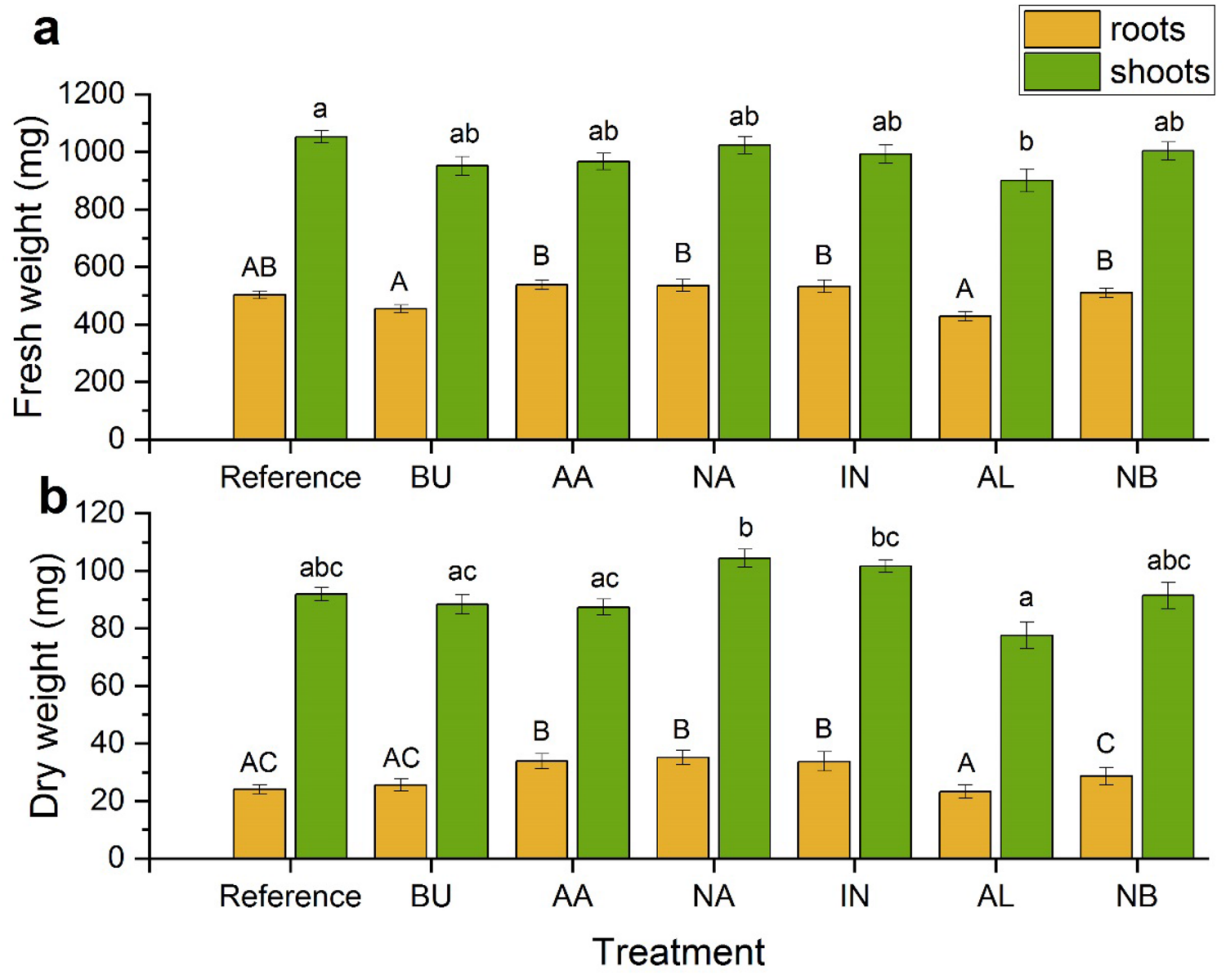
The influence of zinc oxide treatments on fresh (**a**) and dry (**b**) weights (mg) of pea plant as calculated for an average single pea plant after 12 days of contact with ZnO species. Roots are indicated in yellow while shoots are in green. Vertical bars represent standard errors. Distinct letters show the statistically significant difference as calculated with the Tukey’s HSD test, roots and shoots are treated separately. The significance level α = 0.05 was applied.

The seventh series was a reference administered with initial Hoagland solution. Growing media were being replaced every 48 h. Plants were collected after 12 days of zinc administration when (on average) they reached growth stage 15 at the BBCH scale. Shoots and roots were isolated. The latter were washed with demineralized water, and later dried with a filter paper. The lengths of roots and stems were measured (Fig. 2). Weights of the fresh and dry (incubation at 55 °C to the constant weight) shoots and roots were determined (Fig. 3).

### Metals determination in plant material

**Figure 4:**
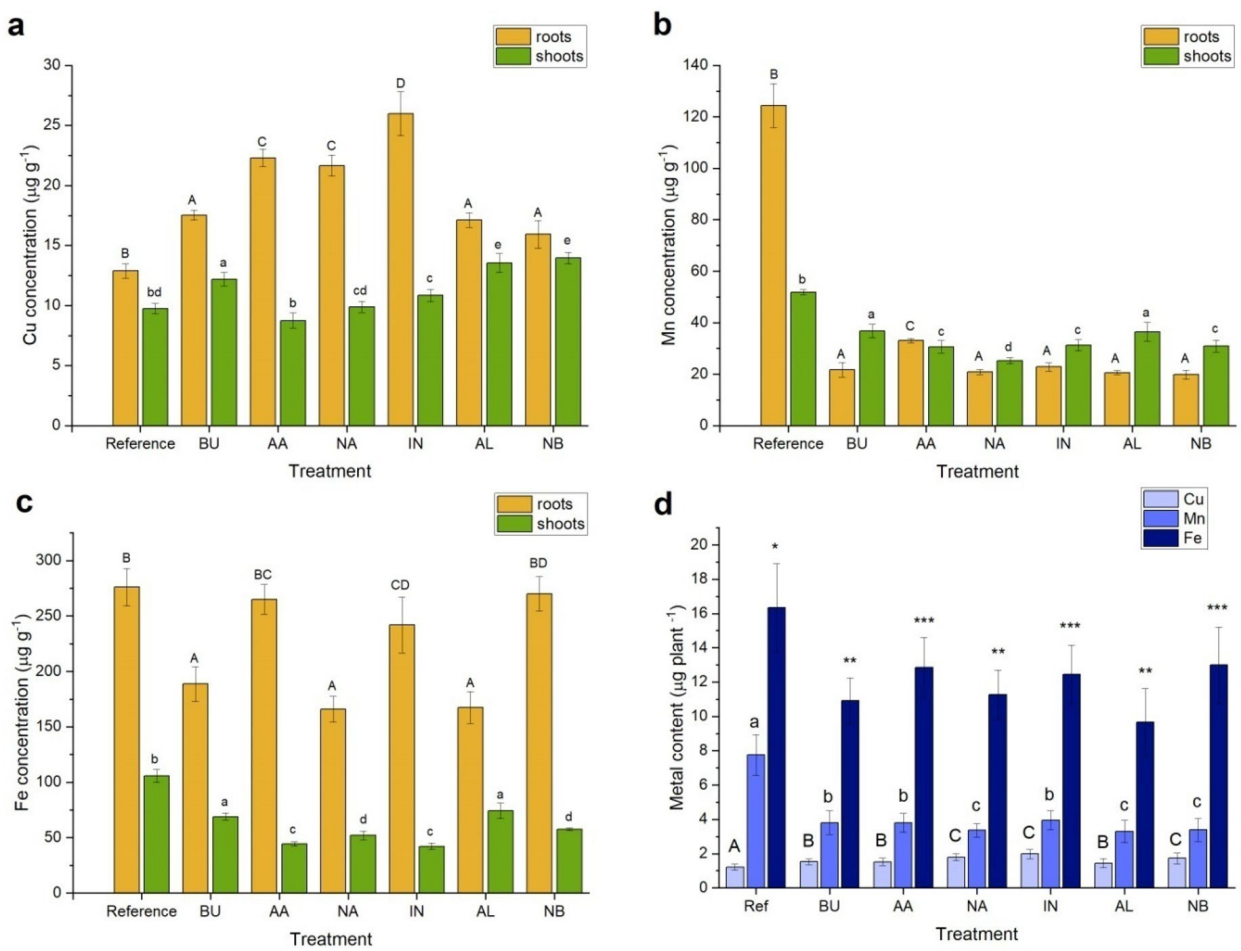
Copper (**a**), manganese (**b**) and iron (**c**) concentrations (µg g^−1^) and total metal content per single plant (µg plant^−1^) (**d**) in sugar pea upon ZnO administration. Roots are indicated in yellow while shoots are in green. Vertical bars represent standard deviation. Distinct letters show the statistically significant difference as calculated with the Tukey’s HSD test, roots and shoots are treated separately. The significance level α = 0.05 was applied.

The content of Cu, Mn, Fe and Zn in plant material (roots and shoots) was determined by the ContrAA 300 atomic absorption spectrometer (Analytik Jena, Jena, Germany) operating in the flame (air-acetylene) mode and equipped with the high resolution continuum radiation source. The carefully weighted plant samples (0.6 g—shoots and 0.3 g—roots) were digested in the mixture of concentrated HNO_3_ and HCl (6:1, v/v) using the Anton Paar Multiwave 3000 closed system instrument^47^. Results are summarized in Fig. 4.

### Tolerance index and translocation factor

The tolerance index (TI) is the ratio of mean roots length determined for plants grown in zinc triggered stress conditions as related to the relevant roots size in a reference treatment^48^. Metal distribution inside the plant constituency was represented by translocation factor (TF) which is the ratio of element concentration in above ground parts of the plant to that in roots^49,50^. Those factors give more precise representation of metal migration than the raw metal contents and are to be assessed altogether. They were already used by us for the pesticide induced heavy metal uptake and accumulation in wheat^51,52^.

### Statistical analysis

All analyses were replicated six times. The initial hypothesis on equal variances of investigated populations were validated with the Bartlett and Hartley tests^53^. Normality of the sample distributions was subsequently proved by the Shapiro–Wilk test^54^. A one-way analysis of variance (ANOVA) as implemented in OriginPro 2016 was used to test the influence of ZnO species on plant growth parameters and Cu, Mn and Fe contents in plants cultivated in Hoagland solutions. The Tukey's honestly significant difference (HSD) post hoc test^55^ was used to compare the differences for mean values in each treatment. Roots and shoots were treated separately, α = 0.05 significance level was used in all computations.

## Results

Root and stem lengths of pea plants cultivated in Hoagland solutions after 12 days of contact with ZnO species are presented in Fig. 2. At the 0.95 probability level, BU and NB treatments yielded the same roots elongation as in the reference sample while the decrease for remaining supplementations was observed. The latter are grouped in two distinct pairs represented by letters B and C as in Fig. 2. The former represents AA and AL while the latter NA and IN supplementations. The TI are organized in a decreasing way: BU = NB > AA = AL > IN = NA (Table 1) and clearly support those observations. Divergent view was seen for stems. Their expansion was induced by BU and NB. The AA, AL and NA generated the same stem growth as the reference while the lowest elongation was determined for IN and NA. The fresh weights of roots upon all treatments (Fig. 3a) are not statistically different than those determined for the reference sample. The influence of nanoparticles migration within the plant is the most clearly visible for the AL which significantly inhibits the stem growth as compared to the reference sample. The dry mass of roots is larger than that observed in the reference sample for AA, NA and IN supplementations only. However, the dry weights of shoots upon all treatments are not statistically different than that of untreated control pea plants.

**Table 1:** Tolerance indices (TI) and translocation factors (TF).

Treatment	TI	TF		
		Cu	Mn	Fe
REF	1.00	0.76	0.42	0.38
BU	0.99	0.70	1.70	0.37
AA	0.91	0.39	0.93	0.17
NA	0.74	0.46	1.20	0.31
IN	0.78	0.42	1.37	0.18
AL	0.91	0.79	1.77	0.44
NB	0.97	0.88	1.56	0.21

Zinc contents in roots and shoots as determined for all treatments accompanied by TF are summarized in Table 2. The highest concentrations in roots and shoots were determined for AA and AL administrations, respectively. The most efficient Zn translocation was observed for the latter (TF = 0.28). The highest TF was observed (TF = 0.81) for the untreated reference sample characterized by low Zn concentration in the Hoagland solution. Zinc migration from root to the upper part of plants was inversely proportional to the Zn concentration in roots (Supplementary Information Fig. S1). The latter may be induced by defence mechanisms which are responsible for the Zn immobilization in vacuoles or cell walls^56–59^.

**Table 2:** Zinc concentrations (µg g^−1^) in roots and shoots of sugar pea (mean ± SD; n = 6) accompanied by translocation factors.

Treatment	Zn concentrations (µg g^−1^)		TF
	Roots	Shoots	
REF	92.6 ± 7.0	75.1 ± 8.7	0.81
BU	17,494 ± 859	2,017 ± 97	0.12
AA	49,854 ± 1,270	950 ± 70	0.02
NA	35,841 ± 2,607	1,101 ± 60	0.03
IN	26,968 ± 2,377	847 ± 55	0.03
AL	14,602 ± 1,069	4,033 ± 497	0.28
NB	21,046 ± 3,033	1,250 ± 198	0.06

The Cu, Mn and Fe concentrations in pea plants are shown in Fig. 4a–c, roots and shoots were treated separately. Those metals accumulation by plants as influenced by ZnO species was evaluated by a one-way ANOVA. The null hypothesis was, whether ZnO supplementation had influenced Cu, Mn and Fe uptake from the Hoagland solution. Calculations clearly showed that all ZnO forms affected heavy metals transfer (Table 3). Additionally, the average metal content as calculated for a whole, single plant is presented in Fig. 4d.

**Table 3:** ANOVA parameters for metal content in *Pisum sativum* L. across seven treatments (a) roots and (b) shoots.

Metal	SS_total_	MS_between_	MS_within_	F	p-value	Test F
**(a)**						
Cu	748.2372	119.0105	0.976409	121.8858	5.78E−22	2.371781
Mn	53,924.7	8,913.534	12.67143	703.4357	5.8E−35	2.371781
Fe	97,655.6	14,665.9	276.0059	53.13618	3.88E−16	2.371781
Zn	9.29E9	1.53E9	3,594,200	425.065	3.52E−31	2.371781
**(b)**						
Cu	154.4547	23.86973	0.321036	74.35208	1.89E−18	2.371781
Mn	2,793.593	433.6063	5.484422	79.06144	7.01E−19	2.371781
Fe	18,071.14	2,910.531	17.37018	167.559	2.81E−24	2.371781
Zn	58,593,022	9,671,712	16,078	601.532	8.74E−34	2.371781

All treatments led to the significant reduction of Mn contents either in roots or shoots as compared to the reference sample. Following the Tukey’s HSD test BU, NA, IN, AL and NB treatments yielded the same Mn levels in roots while the AA prompted higher values. Similarly, in shoots the highest Mn content was observed for the reference sample. The decrease of Mn levels upon the BU and AL supplementations was clearly observed. However, the lowest values were determined for AA, IN, NB and NA treatments. The Fe behaved in a more ambiguous way. The AA and NB did not affect iron levels in roots while substantial decrease was observed for BU, NA and AL supplementations. The more diverse pattern was seen in shoots. The Fe migration for upper parts of plants was hampered by either BU and AL or AA and IN groups of ZnO species. The opposite situation was for the Cu which uptake is stimulated by all ZnO formulations applied. The only exception was observed for shoots of plants treated with AA as well as NA. This picture is also reflected by Cu, Mn and Fe contents as calculated for a single plant and averaged over all species in the pot (Fig. 4d). All values clearly indicate that Mn and Fe uptake are hampered while the Cu uptake is prompted by all forms of ZnO applied.

Migrations of Cu, Mn and Fe from roots to green parts of the plant are represented by translocation factors as summarized in Table 1. The AL and NB treatments prompted the metal accumulation in shoots while reverse effect was observed for Cu and Fe upon AA, NA and IN administrations. Additionally, the manganese translocation from roots to shoots was substantially increased after addition of all applied ZnO species. The Mn uptake by roots was extensively hampered in all those treatments. The latter may follow from the well-recognized Zn/Mn antagonism^60,61^. Manganese is an important cofactor of proteins involved in water splitting. This reaction is crucial for the photosynthesis system II^62^. Therefore, increasing demand for Mn in green parts of the plant accelerates internal Mn transport from roots to shoots.

## Discussion

Interactions of plant roots with nanomaterials are complicated processes and several mechanisms responsible for NPs uptake and further translocation have been identified^63–67^. It is widely accepted that nanoparticulate ZnO may be better absorbed by plants than the bulk form^68^. However, this picture is far from being exhausted and opposite view has also been published by Milani et al.^69^.

There are evidences that the uptake of nanoparticulate ZnO involves dissolution and ionization which may be prompted by the acidic root exudates^70^. However, the direct absorption of pristine NPs cannot be neglected^26,71,72^. For a long time, the relatively low values of size exclusion limits, as determined for plant roots, indicated that large NPs can hardly enter root tissues in a raw form. This statement was challenged by Nair et al.^73^ who pointed out that NPs may induce destruction of the cell wall and enlarge the pore size. The cell wall is a complex cellulose and hemicelluloses matrix stabilized by pectins which are pivots located in the hollow open spaces^74,75^. They are important targets for reactive oxygen species^76^. The latter are harmful by products of stress processes induced by nanomaterials. Wounds in plant roots may be also avenues for large NPs uptake^77^.

Our results indicate that the elevated concentrations of zinc in roots were accompanied by its relatively low contents in upper parts of plants. The dissolved zinc concentrations in Hoagland solutions as determined by the centrifugation–filtration procedure of Landa et al.^45^ and Mukherjee et al.^24^ were below 10% of the total zinc supplementation as ZnO to the growing media [100 mg(Zn) L^−1^]. Notable, the reference, raw solution contained only 0.01 mg(Zn) L^−1^. We therefore hypothesize that to a large extent the ZnO is absorbed by roots in a nanoparticulate form through variety of cell pores. On the contrary to mobile ionic Zn^78^, those nanoparticles are stabilized in root tissues and are not fully available to green parts of the pea plant.

All investigated NPs exhibit a natural propensity to form aggregates in the Hoagland solution. This is indicated by a substantial hydrodynamic diameter as determined by the Dynamic Light Scattering (DLS) measurements and further supported by the low zeta potential values (Supplementary Information Table S1). Similarly to results of Kim et al.^79^, Liu et al.^80^, Lizunova et al.^81^, Cao et al.^82^ the NPs TEM sizes are significantly smaller than those of DLS and suggest soft, dynamic character of micelle aggregation which could easily adopt to pore diameter in the root surfaces and cell walls.

The nonselective apoplastic or selective symplastic pathways are involved in metal uptake by plant roots in either ionic or nanometric forms^83,84^. The latter mechanism strongly depends on transmembrane metal transporting proteins^85^. Opposite, the apoplastic route is correlated with the transpiration^86^. In this study we observed decrease of Mn and Fe contents combined with the Cu level increase in the plant body. We speculate that the former metals are transported via symplastic pathway and compete with Zn^2+^ for similar carriers. On the contrary Cu is accumulated via nonselective apoplastic route. Those mechanisms depend on the carriers concentrations which follow the rate of particular proteins synthesis^87–89^. In particular, the low accumulation of Fe can be related to down regulation of IRT1 and IRT2 iron regulating genes induced by the zinc toxicity^90^. Similar mechanisms as developed by plants to avoid the harmful effects of nanoparticles and involving genes of the IRT family for Cd, Cu, Zn, Co and Mn were also reported^91^.

## Conclusions

Our results unequivocally show that zinc compounds at either molecular or nanoscale levels alter Cu, Mn and Fe uptake and their further migration in *Pisum sativum* L. On the contrary to the last two metals, the Cu content increased in roots upon all treatments applied. Unfortunately, the picture for shoots is not so clear with AA reducing the Cu levels. Additive interactions which either restrain or enhance heavy metals uptake are important indicators of the pollutants migration mechanisms. They are of special value when environmental effects induced by zinc species as present in wastes, urban low emissions and food chain components are to be concerned. Our results are in line with recent publications which report that the nanoparticle activity is a complex issue and extends beyond the well recognized mechanisms of metal ion uptake. Unfortunately, the detailed mechanism of this process has not been fully recognized as yet. Investigations of Adamczyk-Szabela et al.^42^ clearly indicate that nutrients affect zinc uptake but the reverse process i.e. the nutrients uptake upon zinc presence cannot be neglected. In this study we firmly confirm that the latter is important for the plant development.

The steadily increasing abundance of nanomaterials in either water or soil environment may also affect biochemical processes responsible for metal and nutrients uptake by agricultural plant species. This effect deserves more attention and should not be neglected when either hydroponic solutions or soil fertility are at stake. Unfortunately, existing regulations for the plant cultivation originate from the times when anthropogenic nanoparticles were not in a common use. Legislators and their advisors often look at nanomaterials impact on agriculture in an oversimplified way and neglect complexity of metals interactions in either plant or habitat.

## Supplementary information

Supplementary Information
