# Supplementary material for: Additive interactions of nanoparticulate ZnO with copper, manganese and iron in Pisum sativum L., a hydroponic study
Source: Sci Rep. 2020 Aug 11;10:13574. doi: 10.1038/s41598-020-70303-8 (PMC7421903; doi:10.1038/s41598-020-70303-8)
Supplement: Supplementary file 1 — Supplementary Information [file 41598_2020_70303_MOESM1_ESM.pdf]

Supplementary Information for

Additive interactions of nanoparticulate ZnO with copper, manganese and iron in *Pisum sativum* L. A hydroponic study

Elżbieta Skiba*[1], Sylwia Michlewska[2], Monika Pietrzak[1], Wojciech M. Wolf[1]

[1]Lodz University of Technology, Institute of General and Ecological Chemistry, Zeromskiego 116, 90-924 Lodz, Poland

[2] University of Lodz, Faculty of Biology and Environmental Protection, Laboratory of Microscopic Imaging and Specialized Biological Techniques, Banacha 12/16, 90-237 Lodz, Poland

*corresponding author: elzbieta.skiba@p.lodz.pl, +48 42 631 31 23, ORCID: 0000-0001-8678-5256

Table S1. Average TEM particle sizes, hydrodynamic DLS particle sizes and zeta potentials determined in growing media. Standard deviations are given in parenthesis.

Nanoparticle	TEM (nm)	DLS (nm)	Zeta Potential (mV)
AA	58 (18)	205 (10)	- 3.59 (0.69)
NA	13 (3)	503 (83)	- 3.81 (0.48)
IN	71 (21)	377 (37)	- 2.91 (0.08)
AL	61 (20)	242 (25)	- 2.26 (0.43)
NB	51 (18)	93 (6)	- 3.30 (0.29)

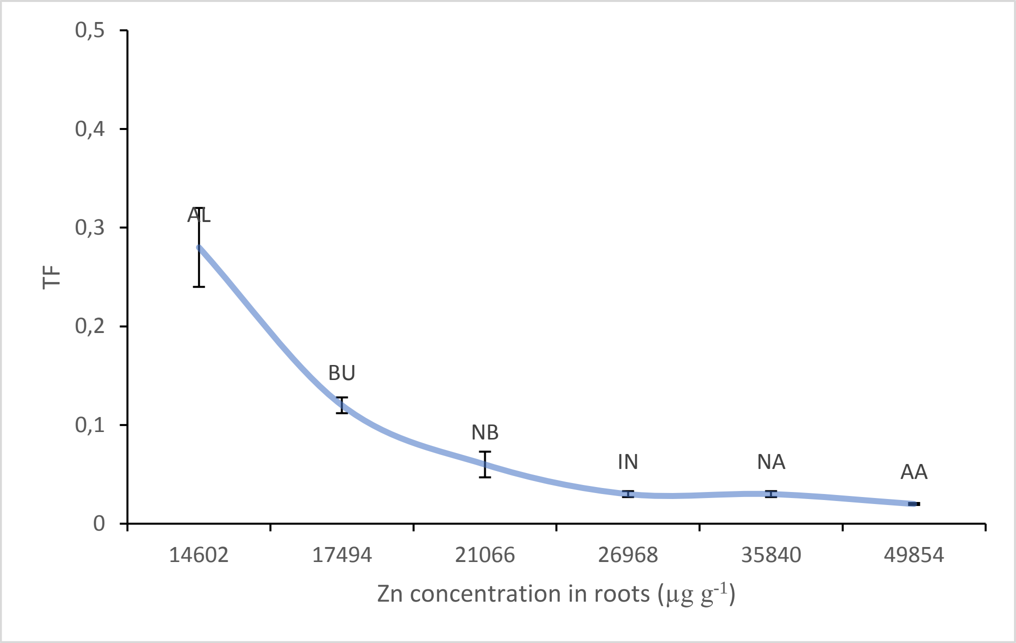

Figure S1. The influence of Zn concentration in roots on its translocation factor for all ZnO supplementation applied. Vertical bars represent standard deviations of TF values.
